# Comparison of Cervical Biportal Endoscopic Spine Surgery and Anterior Cervical Discectomy and Fusion in Patients with Symptomatic Cervical Disc Herniation

**DOI:** 10.3390/jcm13061823

**Published:** 2024-03-21

**Authors:** Seok-Bong Jung, Ishant Gunadala, Nackhwan Kim

**Affiliations:** 1 Spine Center, Jinju Bon Hospital, Jinju-si 52703, Republic of Korea; jsb0079@naver.com; 2 Department of Orthopaedic Surgery, Government Hospital Ahmedabad, Ahmedabad 380049, India; drishantimmanuel@gmail.com; 3 Department of Physical Medicine and Rehabilitation, Korea University Guro Hospital, Seoul 08308, Republic of Korea

**Keywords:** cervical radicular pain, cervical disc herniation, anterior cervical discectomy and fusion, biportal endoscopic spine surgery, pain intensity, blood loss

## Abstract

**Background:** We aimed to analyze the clinical outcomes and effectiveness of cervical biportal endoscopic spine surgery (C-BESS) and anterior cervical discectomy and fusion (ACDF) in patients with symptomatic cervical disc herniation. **Methods:** This study was a retrospective chart review of four-year clinical data involving 318 cases of symptomatic cervical disc herniation, with 156 patients undergoing the ACDF and 162 patients receiving the C-BESS. Preoperative and postoperative one-year data were collected. **Results:** The numeric rating scale and neck disability index showed statistically significant improvement for both ACDF and C-BESS groups. While showing a longer operation time and more blood loss during surgery compared to the ACDF group, the C-BESS group demonstrated a learning effect as the surgeon’s proficiency increased with more cases. There was no significant difference in the postoperative length of hospitalization between the two methods. The subgroup with predominant arm pain revealed the statistical difference in arm pain intensity changes between the two groups (*p* < 0.001). The rates of complication were 2.6% for the ACDF group and 1.9% for the C-BESS group. **Conclusions:** C-BESS and ACDF are effective surgical treatments for patients with symptomatic single-level cervical disc herniation in relieving relevant pain intensities and pain-related disabilities.

## 1. Introduction

Cervical disc herniation (CDH) often leads to cervical radicular pain, which may be accompanied by numbness, tingling, weakness, or other symptoms in the affected arm or hand [1]. The manifestation of dermatomal and myotomal symptoms attributed to a specific cervical disc herniation correlates with the spatial alignment between the nerve root and the pedicle. Radicular pain from cervical disc herniation (CDH) arises not only from mechanical compression but also from inflammatory changes within the disc [2]. The disc’s biomechanical strength depends on the equilibrium between hydrostatic pressure and shear stress. Disruption of this balance, such as during trauma or disc degeneration, can lead to increased radial bulge, potentially causing nerve root or spinal cord impingement [3].

Surgical treatment for cervical radicular pain due to a herniated disc is typically considered when conservative measures such as physical therapy and medication fail to provide relief. The most common surgical intervention for symptomatic CDH is anterior cervical discectomy and fusion (ACDF), which involves removing the affected disc and replacing it with a bone graft and/or metal plate to stabilize the spine [4,5,6]. Numerous studies have shown that ACDF is an effective treatment for cervical radicular pain caused by herniated discs, with most patients experiencing significant pain relief and improvement in function. However, similar to all surgical procedures, ACDF carries risks such as infection, bleeding, postoperative pain, and nerve damage [7]. Cervical arthroplasty, introduced by Reitz, is increasingly favored over ACDF due to its potential to preserve motion and reduce adjacent segment disease [8]. The procedure involves an anterior approach and placement of a prosthesis after discectomy. While various prosthetic designs show promising results, complications such as implant failure and bone loss may arise [9]. Posterior cervical foraminotomy (PCF) can be performed via endoscopic or open techniques [10]. Endoscopic PCF involves a small incision off the midline, followed by nerve root decompression using specialized instruments. While open approaches may cause more postoperative pain, endoscopic PCF typically provides high rates of pain relief [10]. However, concerns exist regarding potential instability after bone resection, particularly without arthrodesis, and PCF may not be suitable for conditions like ossification of the posterior ligament or central hernia [11].

In the last three decades, there has been significant progress in minimally invasive spine surgery to minimize damage to normal spinal structures during procedures and enhance functional outcomes. Endoscopic spine surgery stands out as a notable example. Initially, this approach cautiously addressed posterolateral disc herniations through a transforaminal route, encountering numerous limitations such as restricted surgical instruments, impaired visibility from existing camera systems, high radiation exposure, and a steep learning curve. Nevertheless, recent years have witnessed a swift expansion of endoscopic surgery from lumbar to cervical lesions, propelled by advancements in endoscopic equipment and the introduction of novel surgical techniques [12].

Recently, newer techniques, such as cervical biportal endoscopic spine surgery (C-BESS), which involves the selective removal of the herniated disc by approaching through two ipsilateral portals, have been developed as a surgical option for symptomatic CDH. An endoscope with a high-resolution view and intuitive working instruments are introduced into the portals. A well-trained surgeon then uses specialized instruments to remove the bony structure or herniated disc without affecting the surrounding healthy tissues. Several reports on the technical notes and clinical outcomes of C-BESS have recently been published [13,14].

To the best of our knowledge, no such study compares the clinical outcomes of C-BESS and ACDF, leaving the potential and relative benefits of C-BESS uncertain. We aimed to compare and analyze the clinical findings of two surgical methods in patients with symptomatic CDH.

## 2. Materials and Methods

### 2.1. Study Design

This study is a cross-sectional observational study comparing two surgical methods. After obtaining study approval from the appropriate hospital institutional review board (No. 2021AS0351), we retrospectively reviewed the records for all patients who had undergone ACDF or C-BESS for symptomatic CDH at a single spine center from January 2017 to December 2021. Operation records, inpatient and outpatient clinical charts, and imaging studies were reviewed, with the lead surgeon and surgical team excluded. Confidentiality was maintained for anonymized data.

### 2.2. Population

**Figure 1:**
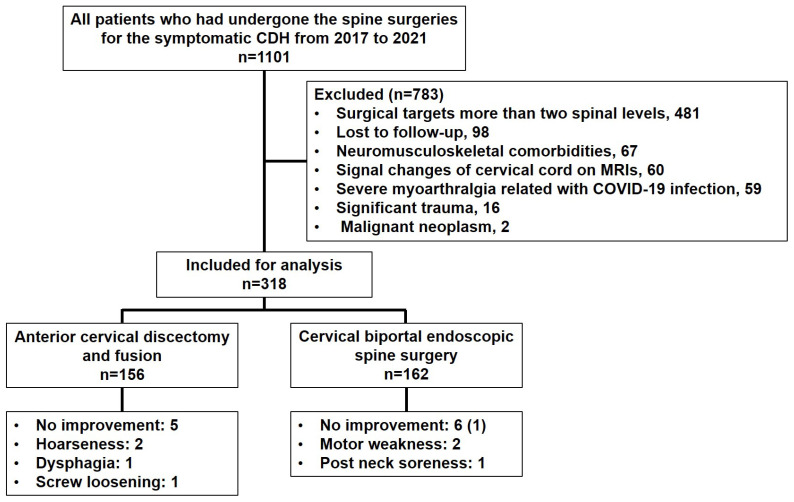
Demographic flowchart of patients undergoing the anterior cervical discectomy and fusion or cervical biportal endoscopic spine surgery who were excluded or included in the analysis. One in six individuals who had presented no improvement in the cervical biportal endoscopic spine surgery group underwent a revision (braces). CDH, cervical disc herniation.

An electronic review of patient charts was conducted to extract the demographic and clinical data of all individuals who met the eligibility criteria. The inclusion criteria were as follows: a patient who has undergone ACDF and C-BESS for symptomatic CDH and an adult over the age of twenty. Patients with any of the following conditions were excluded: symptom duration of less than three months, insufficient evidence of failed conservative management for at least one month, surgical targets of more than two spinal levels (including bilateral lesions), follow-up loss within one year postoperatively, neuromusculoskeletal comorbidities (chronic rotator cuff disorder, peripheral neuropathy, rheumatoid arthritis, or chronic tendinosis), signal changes of the cervical cord on MRIs, severe myalgia and arthralgia related with COVID-19 infection, significant trauma on an upper extremity or cervical spine (whiplash injury, fracture, or severe sprain/strain), and malignant neoplasm diagnosed during the observation period. This left 318 patients eligible for analysis (Figure 1).

### 2.3. Surgical Techniques: ACDF

Under anesthesia, the patient was placed in a supine position on a radiolucent operation table. After the aseptic preparation, a 2-inch incision was made on the left neck side. Tunnels to the spine were created by retracting the muscles, trachea, and arteries. Paraspinal muscles were moved aside to expose vertebral bodies and discs. A thin indicator was located on the affected vertebra and disc under fluoroscopy. A retractor separated adjacent vertebrae, providing surgical space. Subsequently, a discectomy was executed through the customary method with the assistance of a surgical microscope, and the marginal osteophyte was excised using high-speed burrs and angled curettes. The intervertebral foramen was enlarged, and the outer cortical layer was removed. For stability, a peak cage with a demineralized bone matrix was inserted and reinforced with a metal plate. X-rays verified implant positions. Spreader retractors were removed, and incisions were sutured.

### 2.4. Surgical Techniques: C-BESS

**Figure 2:**
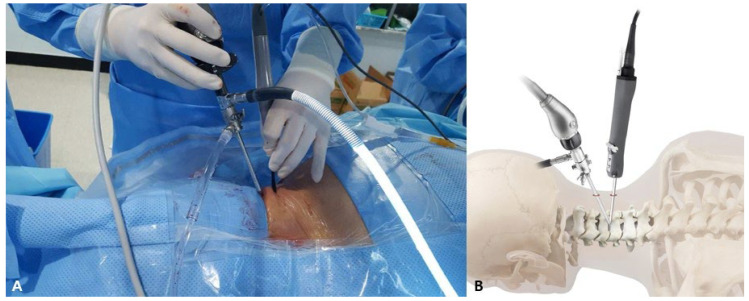
(**A**) Clinical photo of the operative field showing cervical biportal endoscopic spine surgery (C-BESS) for a patient with right subarticular cervical disc herniation at the level of C4/5. The surgeon is positioned on the right side of a prone-positioned patient and manipulates two instruments in both hands. (**B**) Conceptual diagram of C-BESS. The orthopedic arthroscope in the proximal portal approaches the dorsal neck with a target of the V-point on the interlaminar space. The V-point comprises the superior margin of the caudal lamina, the inferior margin of the cranial lamina, and the starting point of the facet joint. The working instrument is inserted into the endoscopic portal on the ipsilateral postal 2–3 cm distal.

Under general anesthesia, patients were placed in the prone position. The face was positioned in a contoured foam pillow with openings for the eyes, nose, and airways while also providing support for the chin and forehead. An incision of approximately 1–1.5 cm was made on the skin, located on the same side as the spinous process midline and at the level of the target interlaminar space. A second incision was made on the skin, positioned 2–3 cm below the location of the first incision. Two portals are involved in the procedure: one for viewing and the other for working. The working one was positioned distally to reduce the extent of laminectomy required (Figure 2A,B). After the scope and electrical ablator were inserted into the two portals, a V-point made of the upper and lower laminas was identified under a pressure-controlled irrigation pump. The target interlaminar area was approached by carefully manipulating the burr to perform a partial laminotomy. Subsequently, a Kerrison punch was used to perform a medial facetectomy. During the procedure, the working tool was angled towards the center and positioned inside the endoscopic view. Unobstructed and pliable movement of the spinal root was confirmed after removing the herniated disc tissue. An adjacent radiofrequency probe was used to perform additional annuloplasty near the perforated annulus to block the escape pathway of the remnants of the nucleus pulposus. The operation of the irrigation pump was stopped, and a thorough examination of the bleeding point was conducted before removing the devices from the two portals. The subcutaneous fascia and skin were sutured after the instruments were removed, and stagnant irrigation saline was used.

### 2.5. Postoperative Care

Under a serial neurological evaluation, the patients were permitted to sit while wearing a soft collar for eight hours postoperatively. If there were no issues with balance while sitting, patients were allowed to attempt walking under close supervision. Within 24 h of surgery, patients underwent either an MRI or CT scan to assess for any potential soft tissue problems, such as the formation of a hematoma or swelling, and to determine whether tissue removal was sufficient. The patients were discharged once their mobility was ensured.

### 2.6. Outcome Evaluation

The collected data included epidemiological information, MRI and plain film findings, clinical information, and functional assessment results. Numeric rating scale (NRS) pain scores, ranging from 0 (no pain) to 10 (worst pain imaginable), and a neck disability index (NDI) on a 0 to 100% scale (higher scores represent increased levels of related disability) were assessed preoperatively and at a follow-up visit scheduled approximately one year after surgery [15]. The total operation time was defined from incision time to closure completion time, which was determined by cross-referencing information from the surgical, anesthesia, and nursing records. The length of hospitalization was defined as the number of days from the date of surgery to the date of discharge. The amount of perioperative blood loss was estimated based on the hematocrit before and after surgery: the estimated blood volume x $\ln\frac{the\;initial\;hematocrit}{the\;final\;hematocrit}$ [16,17]. Among the formulas used to estimate blood loss, Ward’s formula can be calculated based on pre- and postoperative hematocrit levels. The estimated blood volume is determined using Nadler’s method, which takes into account the patient’s gender, surface area, and body mass, simplified per kilogram based on age and gender [18]. For the complication investigation, both inpatient and outpatient medical records underwent a qualitative review. The assessment of complications relied on various indicators: if the NRS revealed less than a 20% recovery from preoperative symptoms, it was categorized as “no improvement”. Additionally, complications were evaluated based on subjective patient reports gleaned from various sources such as progress notes, nursing charts, patient education records, and outpatient medical records.

Subgroups were analyzed for the efficacy of symptomatic features: NECK, a subgroup with initial neck pain greater than arm pain; ARM, a subgroup with initial arm pain greater than neck pain.

### 2.7. Statistics

Power analysis was conducted using a priori sample size calculator for the Wilcoxon–Mann–Whitney test. To reach a power of 95%, a sample size of 156 patients in each group was calculated, with an alpha level of 0.05 and an effect size of 0.42 derived from previously published data [19].

The Shapiro–Wilk test was used to assess the normality of all continuous data. The mean and 95% confidence interval (CI) are reported for normally distributed variables, while the median and interquartile range (IQR) are reported for non-normally distributed variables. The Wilcoxon rank sum test was used to compare demographic data such as age, symptom duration, initial NRS and NDI, operative time, estimated blood loss, and length of hospitalization. The Mann–Whitney U test was used to compare pre- and postoperative data within each group. Categorical data, including sex and target disc level, were analyzed using a chi-square test. The improvement in pain intensity was presented as a NRS ratio of the change in the initial value: $A\;NRS\;ratio=\frac{preoperative\;NRS-postoperative\;NRS}{preoperative\;NRS}$. The Mann–Whitney U test was used to compare the NRS ratio between the subgroups. The learning effect of surgical techniques was analyzed via a cumulative sum control chart (CUSUM), which reveals the continuous summation of differences between the operation time for each case and the mean value of total cases [20]. The formula is as follows: CUSUM = $\sum_{i=1}^{n}\left(X_{i}-M\right)$. *X_i_* indicates the individual value, and *M* indicates the mean value. The null hypothesis was that there was no outcome difference between the ACDF and C-BESS groups. The level of significance was set at *p* < 0.05. Statistical analyses were performed using SPSS (version 25.0; IBM Corp., Armonk, New York, NY, USA).

## 3. Results

### 3.1. Demographic Characteristics

The diagnosis and decision of surgical intervention were performed by a single physician, who provided the information about both ACDF and C-BESS procedures to the patients only if a single level of unilateral lesion was indicated. Subsequently, the final decision on the type of surgery was made on the patient’s own initiative after consultation with the surgical team.

The data of the 318 eligible patients were included in this study: 156 patients with ACDF (ACDF group) and 162 patients with C-BESS (C-BESS group). As shown in Table 1, there were no statistical differences in sex, age, symptom duration, initial NRS, NDI scores, or target disc level between the two groups.

### 3.2. Outcome Measures

Statistically significant improvements in pain and disability were observed before and one year after the procedure based on serial NRS and NDI assessments (Table 2).

**Figure 3:**
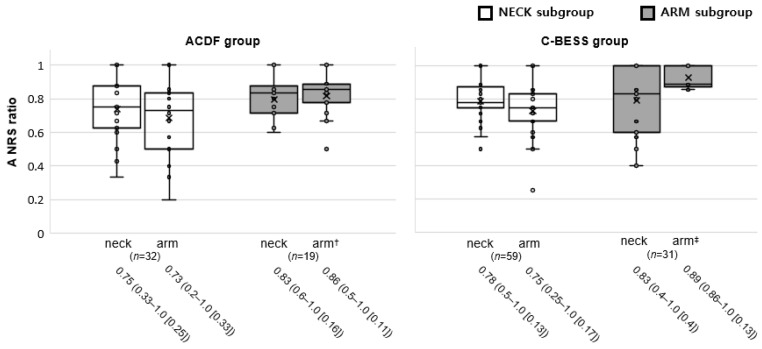
Analysis of NECK and ARM subgroups’ outcomes according to the dominance of neck and arm pain. A NRS ratio represents the ratio of the interval change to the initial value. The data are expressed in boxplots, which show the values of the NECK subgroup as a white box and the ARM subgroup as a gray box. Box plots summarize data by showing the minimum, maximum, median, and quartiles. The box represents the middle 50% of the data (from Q1 to Q3), with the line inside denoting the median. Whiskers extend to the minimum and maximum values, excluding outliers. Outliers are shown as individual points (blank dots) beyond the whiskers, highlighting extreme values. They are helpful for comparing distributions, detecting outliers, and understanding data spreads and central tendencies succinctly. The ARM subgroups (gray boxes) revealed the statistical difference in arm pain intensity improvement (arm NRS ratio) between the ACDF (arm†) and C-BESS (arm‡) groups (*p* < 0.001). The data below are median (range [IQR]).

The changes in the NRS (NRS ratio) of the subgroups in both groups were analyzed. The arm and neck NRS ratio of the NECK subgroup between ACDF and C-BESS groups was statistically insignificant (respectively, *p* = 0.2 and 0.3). The neck pain of the ARM subgroups, with predominant arm pain complaints, showed no statistical difference in NRS changes between the ACDF and C-BESS groups (*p* = 1.0). However, in the ARM subgroups, the interval changes in the arm NRS revealed statistical significance between the ACDF and C-BESS groups (*p* < 0.001) (Figure 3).

**Figure 4:**
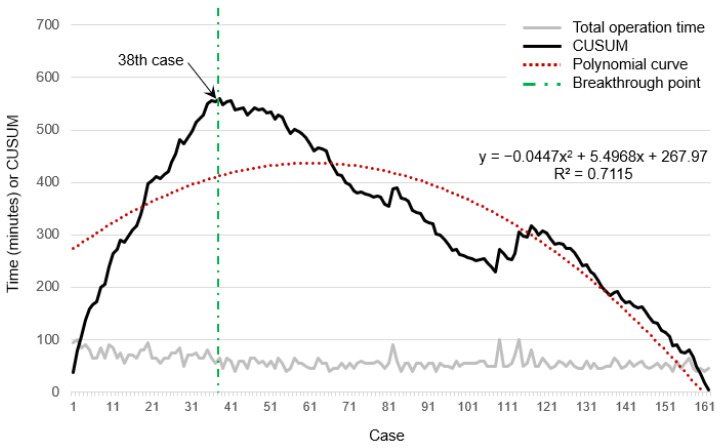
Total operation time and a cumulative sum control chart (CUSUM) curves of the group. The CUSUM curve (black line) shows the continuous summation of differences between the operation time for each case and the mean value of total cases. The red dotted line represents the curve of the best fit for the plot [a second-order polynomial with equation CUSUM = $-0.04\times {\left(case\;number\right)}^{2}+5.49\times \left(case\;number\right)+267.97;\;R^{2}=0.71$]. The green dotted line indicates the breakthrough point (38th case). The gray line represents the actual operation time of each patient.

As shown in Figure 4, the CUSUM based on the total operation time exhibited an initial steep incline 1st the first case to the 38th case, followed by a subsequent gradual decline with a relatively plateau profile.

### 3.3. Perioperative Data

There was a statistically significant difference in the operative time and estimated blood loss between the two surgical methods. But, the median length of hospitalization was not significantly different (Table 3).

### 3.4. Complications

Cases with poor outcomes and complications are presented in Table 4.

In the ACDF group, four significant complications emerged. Two patients complained of hoarseness during a few weeks after surgery. A 64-year-old male faced postoperative dysphagia, experiencing intermittent aspiration during liquid food intake for three weeks. In a separate case, a 73-year-old female exhibited anterior plate screw loosening during the six-month follow-up, requiring revisions.

A 54-year-old male patient in the C-BESS group experienced a decrease in ipsilateral motor function postoperatively, specifically weakness during shoulder abduction and elbow flexion. The patient’s initial Medical Research Council (MRC) grade 4 rating was downgraded to a MRC grade 2. Postoperative MRI confirmed dorsal epidural fluid retention in the cervical spine. The patient’s condition improved to grade 4 after three months of conservation, and an electromyographic study postoperatively one month later showed no abnormalities. Another 68-year-old male patient complained of grade 3 ipsilateral motor weakness in the lower extremity immediately after undergoing C-BESS. Thoracic epidural fluid retention without any signal change in the spinal cord was observed on the postoperative MRI. The long tract signs were unclear, and symmetric waves within normal latencies were found in a somatosensory-evoked potential study. He was able to walk one week postoperatively with minimal assistance but complained of weakness for three months. A 59-year-old male patient who had undergone C-BESS was reconsidered due to poor outcome. The MRI postoperative four months was performed, and revision was decided because it was judged to be insufficient decompression.

## 4. Discussion

There are currently no agreed criteria for diagnosing cervical radicular pain from CDH. Limited evidence exists for clinical tests and patient interviews [21]. Specific symptoms like shoulder or scapular pain and symptom reduction with neck movements were identified, but they are not exclusive to cervical radicular pain. More research into diagnostic accuracy is needed. While we clinically identified the radicular pain originating from the relevant level attributed to symptomatic CDH, a single level was determined through imaging, and the symptomatic correlation was inferred based on neuroanatomical understanding. While this approach may contribute to the patient’s diagnostic evaluation in clinical practice, it may not be deemed sufficient. Nonetheless, these criteria have the potential to exclude cases where the treatment target site is ambiguous, or the pathology exhibits a weak association with symptoms among the prevalent causes of cervical radicular pain.

The analysis of results showed that ACDF and C-BESS emerged as effective and safe treatments for patients experiencing symptomatic single-level CDH. Significantly improved pain intensity and decreased pain-related disability were observed, with relatively low complication rates. C-BESS, while showing a longer operation time and more blood loss on average during surgery compared to ACDF, demonstrated a learning effect as the surgeon’s proficiency increased with more cases. There was no significant difference in the postoperative length of hospitalization between the two methods. C-BESS may offer a more favorable therapeutic outcome than ACDF in a patient with upper extremity pain more than posterior neck pain. In the realm of research on surgical strategies for the relatively prevalent CDH, there is a notable scarcity of studies that specifically compare and analyze ACDF and C-BESS. The scarcity is particularly evident in exploring the clinical application process of the recently introduced C-BESS method among spinal specialists.

ACDF is a “gold standard” surgical procedure traditionally performed to address issues in the cervical spine, particularly involving herniated or degenerated discs [22]. Nevertheless, the persistent consideration of posterior approaches and minimally invasive techniques stems from the fact that accumulated complication reports of ACDF have primarily focused on anatomical structures associated with anterior approaches [23]. Furthermore, there are accounts suggesting that a posterior approach, facilitating direct access to the intervertebral foramen, offers a more favorable long-term prognosis [24]. However, instead of establishing a hierarchy among surgical methods, the prior literature underscores the value of embracing a diversity of surgical options, empowering surgeons to offer a nuanced and personalized approach to patient treatment.

In recent years, a novel treatment option for cervical spine disease, C-BESS, has been introduced. The use of two portals, endoscopic and working portals, in C-BESS provides a clear and safe surgical field, enabling surgeons to perform decompression or discectomy with adequate visualization. The advantages of C-BESS may be multifaceted, including smaller incisions resulting in less damage to the paraspinal musculature, improved surgical visualization, and a shorter recovery time for the patient [25]. Perioperative blood loss can be notable due to the thicker muscle layer encountered during the posterior approach compared to the anterior approach. While this study’s results indicated a larger amount of estimated blood loss, it did not reach a level necessitating transfusion.

Foraminal pathology can be treated directly using posterior approaches. Specifically, the partial removal of the lamina and facet on the dorsal aspect can enhance the success rate of decompression, irrespective of the ease of removing the space-occupying lesion within the ventral epidural space. Our results indicate that the subgroup with predominant arm pain, likely indicative of radiculopathy or radiculitis, revealed the statistical difference in arm pain intensity changes between the ACDF and C-BESS groups. Additionally, the number of patients (one case) with less than a 20% reduction in arm NRS after C-BESS was relatively small compared to those (four cases) after ACDF. An aspect of concern revolves around the opening of the intervertebral foramen relevant to ameliorating arm pain. These results can be concluded in two points: First, the efficacy of C-BESS on the appropriate decompression of the affected cervical root in patients with predominant arm pain due to CDH; second, statistical limitations could possibly show false-positive results due to the enrollment of patients with a positive predictor into the C-BESS group [26].

Persistent pain is a common surgical failure resulting from insufficient decompression. To avoid the ongoing pain, it is imperative to perform broad decompression: lateral laminectomy and facetectomy. However, it is crucial to balance the need for sufficient decompression while preserving the facet joint to maintain segmental stability [27,28]. With the surgical techniques of C-BESS, achieving higher facet reservation and performing fewer osteotomies is possible by placing the instrument under the lamina [29].

Because the clinical application of C-BESS was introduced recently, we analyzed the learning effect on the surgeon. The total operation time initially surpassed the average, gradually declining to the average starting from the 38th case. Subsequently, a noticeable decrease from the average has been observed since the 55th case. The presumption was that the efficacy of surgical techniques, attributed to the learning curve, led to holistic clinical advantages without prolonged time delays. The cut-off point of the learning curve was reported to be selected as 24 cases in unilateral biportal endoscopic spinal surgery for lumbar disc herniation [30]. Concerning the learning curve for percutaneous transforaminal endoscopic lumbar spinal surgery, the early literature suggested a cut-off point of approximately 20 cases [31]. The incidence of complications such as dura or root injury, facet violation with resultant instability, and vascular injury can occur if the surgeon is unfamiliar with the procedure [32]. A well-trained surgeon in C-BESS can expect better surgical outcomes.

The identified complications associated with ACDF in this study are consistent with those documented in earlier cases, and notably, the incidence rate in single-level surgeries closely corresponds to that reported in existing research [4,33]. While the occurrence of complications with C-BESS is comparable to that of ACDF, it is noteworthy that the nature of these complications tends to be relatively more serious. Following a posterior biportal approach and foraminal opening, utilizing an irrigation system with sustained fluid pressure is imperative. This is crucial for ensuring endoscopic visibility, preventing microbleeding, and eliminating necrotic tissues. However, it is essential to avoid excessive fluid retention in the epidural space, maintaining the irrigation system pressure at around 30 mmHg [13].

This study has several limitations. First, the study’s generalizability may be affected by several factors, including the small sample size and the fact that it was conducted by a single surgeon at a single center. These factors have the potential to introduce bias into research outcomes. To mitigate this, we thoroughly evaluated patient-reported outcomes across multiple domains, ensuring the exclusion of the surgeon from this process. Second, the follow-up period was relatively short, and a longer follow-up period is needed to assess potential complications, such as reoperation and device failure. Finally, some potential confounding factors, such as smoking, physical activity, and medication history, were not included in the analysis because of the retrospective design of the study. Further prospective studies with larger sample sizes and longer follow-up periods are required to confirm these findings and address these limitations [34].

## 5. Conclusions

In conclusion, C-BESS and ACDF are effective surgical treatments for patients with symptomatic single-level CDH. For ACDF and C-BESS as the patient-selected surgical methods under the given information, we suggested the evidence of retrospective study as an eligible method for improving the pain intensity and pain-related disorders.

## Figures and Tables

**Table 1:** The baseline characteristics of both groups. Values are median (range) and [IQR].

Variables	ACDF ^1^ (n = 156)	C-BESS ^2^ (n = 162)	*p*-Value
Sex, male: female	101: 55	113: 49	0.3
Age, years	52 (34–83), [15]	54 (29–81), [16]	0.4
Symptom duration, months	12 (6–60), [8]	12 (6–52), [12.5]	0.8
Initial neck NRS ^3^	8 (4–9), [1]	8 (5–9), [2]	0.8
Initial arm NRS	7 (4–9), [2]	7 (4–9), [2]	0.8
Initial NDI ^4^, %	42 (20–78), [14]	40 (20–78), [26]	0.1
Herniation levels, %			
C3/4	1.9, n = 3	3.7, n = 6	0.1
C4/5	19.2, n = 30	17.9, n = 19	
C5/6	60.3, n = 94	52.5, n = 85	
C6/7	18.6, n = 29	21.6, n = 35	
C7/T1	0.0, n = 0	4.3, n = 7	

^1^ ACDF group underwent the anterior cervical discectomy and fusion surgery; ^2^ C-BESS group underwent the cervical biportal endoscopic spine surgery; ^3^ NRS, numeric rating scale; ^4^ NDI, neck disability index.

**Table 2:** Results of patient-reported outcome measures, preoperative and postoperative one year, in both groups. Values are mean (range), standard deviation (SD), and (95% confidence interval).

Group/Variables	Preoperative	Postoperative One Year
ACDF ^1^		
neck NRS ^3^	7.3 (5–9), SD 1.0, (7.1 to 7.5)	1.6 (0–4), SD 1.0, (1.4 to 1.8)
arm NRS ^3^	7.0 (4–9), SD 1.5, (6.7 to 7.2)	1.5 (0–4), SD 0.9, (1.4 to 1.7)
NDI	42.2 (20–78), SD 14.2, (43.0 to 47.5)	14.3 (4–38), SD 8.2, (13.0 to 15.6)
C-BESS ^2^		
neck NRS ^3^	7.2 (5–9), SD 1.2, (7.1 to 7.4)	1.4 (0–4), SD 0.9, (1.3 to 1.5)
arm NRS ^3^	6.9 (4–9), SD 1.5, (6.7 to 7.1)	1.3 (0–4), SD 0.8, (1.1 to 1.4)
NDI ^4^	42.9 (19–80), SD 15.1, (40.6 to 45.3)	14.9 (4–36), SD 8.8, (13.5 to 16.2)

^1^ ACDF group underwent the anterior cervical discectomy and fusion surgery; ^2^ C-BESS group underwent the cervical biportal endoscopic spine surgery; ^3^ neck and arm NRS, numeric rating scale about regional symptoms of neck and arm, respectively; ^4^ NDI, neck disability index.

**Table 3:** Perioperative data (n = 318). Values are median (range) and [IQR].

Variables	ACDF ^1^ (n = 156)	C-BESS ^2^ (n = 162)	*p*-Value
Operative time, minutes	35 (30–90) [10]	55 (40–100) [15]	<0.001
Estimated blood loss, mL	78.6 (13.4–190.4) [29.7]	88.5 (45.9–205.3) [21.1]	<0.001
Length of hospitalization, days	2 (1–7) [1]	3 (1–8) [1]	0.4

^1^ ACDF group underwent the anterior cervical discectomy and fusion surgery; ^2^ C-BESS group underwent the cervical biportal endoscopic spine surgery.

**Table 4:** Number of patients with poor outcomes and complications.

Variables	ACDF ^1^ (n = 156)	C-BESS ^2^ (n = 162)
neck NRS ratio ^3^ ≤ 0.2 ^4^	1	5
arm NRS ratio ^3^ ≤ 0.2 ^4^	4	1
Complications [%] (details)	4 [2.6] (hoarseness 2, dysphagia 1, screw loosening 1)	3 [1.9] (motor weakness 2, muscle soreness 1)
Revision	1	1 ^5^

^1^ ACDF group underwent the anterior cervical discectomy and fusion surgery; ^2^ C-BESS group underwent the cervical biportal endoscopic spine surgery; ^3^$\;\mathrm{NRS}\;\mathrm{ratio}=\frac{\mathrm{preoperative}\;\mathrm{NRS}\;-\mathrm{postoperative}\;\mathrm{NRS}}{\mathrm{preoperative}\;\mathrm{NRS}}$. ^4^ The “no improvement” was defined as the NRS ratio revealed less than 0.2. ^5^ For revision of the patient who underwent C-BESS, ACDF was performed in the same target level.

## Data Availability

The data that support the findings of this study are available upon request from the corresponding author. The data are not publicly available due to privacy or ethical restrictions.
